# Muscle mass and physical function in patients with bladder cancer—Data from a prematurely terminated prospective cohort study

**DOI:** 10.3389/fresc.2022.942475

**Published:** 2022-10-06

**Authors:** Lise Høj Omland, Gunn Ammitzbøll, Cecilia Margareta Lund, Henriette Lindberg, Susanne Oksbjerg Dalton, Charlotte Suetta, Helle Pappot

**Affiliations:** ^1^ Department of Oncology, Copenhagen University Hospital, Rigshospitalet, Copenhagen, Denmark; ^2^ Survivorship and Inequality in Cancer, Danish Cancer Society Research Center, Copenhagen, Denmark; ^3^ Department of Clinical Oncology and Palliative Care, Danish Research Center for Equality in Cancer, Zealand University Hospital Næstved, Næstved, Denmark; ^4^ Department of Medicine, Copenhagen University Hospital, Herlev and Gentofte, Copenhagen, Denmark; ^5^ Copenhagen Center for Clinical Age Research, CopenAge, University of Copenhagen, Copenhagen, Denmark; ^6^ Department of Clinical Medicine, Faculty of Health and Medical Sciences, University of Copenhagen, Copenhagen, Denmark; ^7^ Department of Oncology, Copenhagen University Hospital, Herlev and Gentofte, Copenhagen, Denmark; ^8^ Department of Geriatric and Palliative Medicine, Copenhagen University Hospital, Bispebjerg, Copenhagen, Denmark

**Keywords:** bladder cancer, sarcopenia, physical performance, anticancer treatment adherence, frailty

## Abstract

**Background:**

Patients with bladder cancer (BC) have a high prevalence of comorbidity and low adherence to systemic anticancer treatment but it is unknown whether this is associated with sarcopenia.

**Objective:**

We aimed to investigate if the sarcopenia-defining parameters (muscle strength, muscle mass and physical performance) were associated with reduced adherence to systemic anticancer treatment in patients with BC, and if these muscle domains changed during treatment.

**Methods:**

Patients >18 years of age with BC referred for chemotherapy or immunotherapy at Department of Oncology, Rigshospitalet, Denmark were eligible for study inclusion. Measurements were performed before treatment initiation and within one week after treatment termination, and consisted of assessments of muscle strength, muscle mass, and physical performance. Data was compared with thresholds outlined by the European Working Group on Sarcopenia in Older Patient's (EWGSOP2) guidelines and a healthy, age-matched Danish cohort.

**Results:**

Over a period of 29 months, we included 14 patients of whom two completed follow-up measurements. The recruitment rate was <50% of planned due to logistics and Covid-19 related limitations. Consequently, a decision to prematurely terminate the study was made. No patients fulfilled EWGSOP2 criteria for sarcopenia, but the majority had reduction in one or more muscle domains compared to healthy, age-matched individuals. The majority of patients had poor treatment tolerance, leading to dose reductions and postponed treatments.

**Conclusions:**

In this prematurely terminated study, no patients fulfilled EWGSOP2 criteria for sarcopenia, yet, most patients were affected in one or more muscle domains and the majority had compromised treatment adherence.

## Introduction

Bladder cancer (BC) is the 10th most common cancer worldwide and each year, 2000 patients are diagnosed with the disease in Denmark (1, 2). Patients with T2–4aN0M0 BC have potentially curable disease and can be offered neoadjuvant chemotherapy (NAC) prior to radical cystectomy; patients with locally advanced, unresectable or metastatic disease receive life-prolonging treatment in the form of chemotherapy or immunotherapy (3). Patients with BC are generally considered a frail patient group characterized by high prevalence of comorbidity, high rate of hospitalization during treatment and only ≈50% of patients being able to complete planned anticancer treatment (4, 5). The reasons for frailty in patients with bladder cancer may be multiple but sarcopenia could be a partial explanation (6).

During the last decades, the field of sarcopenia research has evolved dramatically. While previously defined solely by low muscle mass, muscle strength and physical performance are now generally accepted parts of a more comprehensive definition of sarcopenia (7). According to the European Working Group on Sarcopenia in Older Patient's (EWGSOP2) guidelines, sarcopenia is defined as the presence of low muscle strength in addition to low muscle mass, which is typically assessed by dual energy x-ray absorptiometry (DXA) or bioimpedance analysis (BIA) (8). If co-presenting with reduced physical performance, sarcopenia is considered severe (8).

Studies across different cancer types have demonstrated that patients with cancer often present with reduced muscle strength, muscle mass and physical performance compared to healthy age-matched individuals and that reductions in these muscle domains are associated with reduced survival and increased risk of complications following anticancer treatment (9–12). Yet, in patients with cancer, muscle strength and performance are not routinely assessed and sarcopenia (reduced muscle mass) is mainly diagnosed retrospectively based on computed tomography (CT) scans of the abdomen by quantifying the muscle area in a cross-sectional scan typically at the L3 level (13).

In a recent systematic review, sarcopenia (reduced muscle mass) was found in 55%–69% of patients with BC before initiating NAC and a decline in muscle mass of 2.6%–6.4% during the course of NAC was demonstrated (14). However, development of and/or changes in preexisting sarcopenia in patients with BC receiving systemic anticancer treatment have never been evaluated in a prospective study. Further, the impact of sarcopenia on adherence to systemic anticancer treatment has not been investigated in patients with BC. Moreover, despite the EWGSOP2 guidelines mainly being aimed at diagnosing age-related sarcopenia (primary sarcopenia), we hypothesized that including assessment of muscle strength and physical performance would add important information in patients with BC (4, 5).

The aim of the GESICA study was therefore to investigate if reduced muscle strength, muscle mass and physical performance were associated with reduced adherence to systemic anticancer treatment in patients with BC (including cancer in the renal pelvis/ureter). Further, we wanted to investigate if these muscle domains changed during systemic anticancer treatment.

## Material and methods

The GESICA study was designed as an exploratory, prospective cohort study, and therefore no formal power calculations were made prior to initiation of the study. Yet, recruitment of at least 30 patients with BC referred to Department of Oncology, Copenhagen University Hospital, Rigshospitalet, over a two-year period was expected. Patients were eligible if they were ≥18 years of age, had Eastern Cooperative Oncology Group Performance Status (ECOG PS) between 0 and 3 and were scheduled for either NAC, first-line (1 L) or second-line (2 L) chemotherapy, or 1 L or 2 L immunotherapy. Patients were included at the initial visit at the oncology department. Baseline measurements were scheduled to be performed before starting treatment, follow-up measurements within one week after end of treatment.

At both time points, measurements consisted of assessment of muscle strength [hand-grip-strength (HGS) and 30-seconds sit-to-stand-test (30 sSST)], muscle mass by both DXA and BIA (BIA measurements not reported), and physical performance [10-meter gait speed (GS)].

We defined sarcopenia and reductions in muscle domains using the EWGSOP2 cut-off values and as not all patients were older than 65 years, we also compared with data from a healthy, age-matched Danish cohort (7, 8). According to EWGSOP2, reduced HGS is <27 kg for men and <16 kg for women, reduced muscle mass [appendicular skeletal muscle mass divided by height squared (ASM/m^2^)] is <7.0 kg/m^2^ for men and <5.5 kg/m^2^ for women, and reduced GS is ≤0.8 m/s for both sexes (8). Using the Danish comparison cohort, values more than one and more than two standard deviations below mean were considered reduced and severely reduced, respectively.

From the electronic medical records (EMRs), information on disease, ECOG PS, medical history and systemic anticancer treatment were obtained. The study protocol was approved by the Regional Ethical Committee (no. 75,803) and by the data protection registry (no. P-2019-800), and registered in clinicaltrials.gov (no. NCT04144270). All patients gave written informed consent before attending the study.

## Results

From 26 July 2018 to 8 January 2021, a total of 14 out of 30 planned patients were enrolled in the GESICA study, corresponding to approximately one patient every second month and less than 50% of the number planned. Of the 14 patients included, only two patients completed follow-up measurements (data not shown), and we therefore decided to terminate the study in January 2021. The main reasons for the low recruitment rate and follow-up assessments were logistic challenges and restrictions including decommissioning of test facilities due to the Covid-19 pandemic.

Patient characteristics, disease specifications and treatment are presented in Supplementary Table S1. In brief, 57% of patients were male and the median age was 74 years. The majority (57%) had ECOG PS 1; the remaining had ECOG PS 0. Baseline measurements of muscle strength, muscle mass and physical performance for men and women and for each patient are shown in Tables 1, 2, respectively.

**Table 1:** Baseline measurements for patients (*n* = 14) in the GESICA study, Copenhagen, 2018–2021.

Baseline measurements	Men			Women		
	Mean/n	Standard deviation	Range	Mean/n	Standard deviation	Range
Anthropometrics						
Height (cm)	172.9	10.0	151.5; 186	166.4	7.6	155.5; 175
Weight (kg)	84.5	18.3	60.2; 115.1	71.5	15.1	59.1; 98.4
Waist circumference (cm)	104.4	12.8	92.3; 125	94.1	12.1	83; 112
Hip circumference (cm)	97.5	7.5	87.5; 108.5	100.2	10.3	5.2
BMI (kg/m^2^)	28.0	4.0	22.4; 35.1	26	5.2	22.3; 33
Lean mass (DXA)						
TLM (kg)	54.6	10.1	37.3; 64.5	41.3	4.7	36.2; 49.4
ASM (kg)	24.1	4.9	16.0; 29.1	17.9	2.7	16.6; 22.8
ASM/height^2^ (kg/m^2^)	8.0	1.1	6.2; 9.3	6.5	0.9	5.7; 7.7
Muscle strength and physical function						
Hand grip strength, dominant hand (kg)	36.8	9.4	22.5; 53.5	28.2	6.4	21.4; 38.8
Gait speed, habitual speed, 10 mWT (m/s)	1.2	0.3	0.8; 1.7	1	0.3	0.6; 1.4
Gait speed, maximal speed, 10 mWT (m/s)	1.8	0.4	1.3; 2.5	1.4	0.4	0.9; 2
30 sSST, repetitions (n)	14.3	4	9; 19	12.3	3.1	8; 17

BMI, body mass index; DXA, dual-energy x-ray absorptiometry; TLM, total lean mass; ASM, appendicular skeletal muscle mass; ASM/height^2^, appendicular skeletal muscle mass adjusted by height squared; 10 mWT, 10-meter walking test; 30 sSST, 30-seconds sit-to-stand-test.

**Table 2:** Baseline measurements for each patient (*n* = 14) in the GESICA study, Copenhagen, 2018–2021. Muscle function assessments are compared to EWGSOP2 cut-off values (status in parentheses) and to healthy, age-matched Danish individuals (status in color: green = normal, yellow = >1 SD below mean, red = ≥2 SD below mean) (7, 8). The EWGSOP2 guidelines do not provide reference values on 30 sSST.

ID	Sex	Age	HGS^a,b^	30 sSST	ASM/height^2^^c^	GS^d,e^
1	M	≥80	37.2 (normal)	17	8.7 (normal)	1.2 (normal)
2	F	70–79	24.7 (normal)	17	7.7 (normal)	1.1 (normal)
3	M	≥80	24.7 (reduced)	10	7.0 (normal)	0.8 (reduced)
4	F	60–69	21.4 (normal)	10	5.8 (normal)	0.6 (reduced)
5	M	70–79	31.1 (normal)	19	7.1 (normal)	1.1 (normal)
6	M	70–79	28.9 (normal)	15	9.2 (normal)	1.2 (normal)
7	F	60–69	38.8 (normal)	14	6.1 (normal)	1.2 (normal)
8	M	70–79	38.0 (normal)	12	6.2 (reduced)	1.2 (normal)
9	M	60–69	45.2 (normal)	–^f^	8.6 (normal)	1.4 (normal)
10	M	60–69	42.8 (normal)	9	8.8 (normal)	1.3 (normal)
11	F	60–69	29.6 (normal)	13	7.4 (normal)	1.4 (normal)
12	M	50–59	53.6 (normal)	18	8.4 (normal)	1.7 (normal)
13	F	≥80	23.6 (normal)	8	5.7 (normal)	0.8 (reduced)
14	F	70–79	31.3 (normal)	12	6.1 (normal)	1.1 (normal)

HGS, Hand-grip-strength; 30 sSST, 30-seconds sit-to-stand-test; ASM/height^2^, appendicular skeletal muscle mass adjusted by height squared; GS, gait speed.

^a^ Cut-off values HGS according to EWGSOP2 guidelines: 27 kg for men, 16 kg for women.

^b^ Dominant hand, best of three assessments used.

^c^ Cut-off values ASM/height^2^ according to EWGSOP2 guidelines: <7.0 kg/m^2^ for men, <5.5 kg/m^2^ for women.

^d^ Cut-off values GS according to EWGSOP2 guidelines: ≤0.8 m/s for both men and women.

^e^ Habitual gait speed.

^f^ Assessment not performed.

According to EWGSOP2, no patients had sarcopenia. However, one patient (7%) had reduced HGS, one patient (7%) had reduced muscle mass and three patients (23%) performed below the cut-off value for GS (Table 2). When compared to healthy, age-matched individuals, one out of 14 patients (7%) could be classified as having severe sarcopenia. Further, four patients (29%) had reduced HGS, three of 13 patients (23%) had reduced performance in 30 sSST, two patients (14%) had reduced muscle mass, and nine of 14 patients (64%) had reduced GS (Table 2).

From reviewing the EMRs of participating patients, we found that the majority completed planned anticancer treatment, yet 10 patients (71%) had dose reductions and eight patients (57%) had one or more treatment cycles delayed due to various complications (Supplementary Table S2).

## Discussion

Although this prospective cohort study on sarcopenia in BC was prematurely terminated due to low recruitment rate, we were able to collect important information on several muscle parameters in this patient group.

Notably, none of the included patients fulfilled the EWGSOP2 criteria for sarcopenia, despite the vast majority of patients (64%) having reduced physical performance (GS) either alone or in combination with reduced muscle strength (HGS or 30 sSST) or muscle mass compared to healthy, age-matched individuals (7, 8). All these patients either had premature termination of anticancer treatment, dose reductions or treatment delays. Although this was also observed among patients with normal muscle function assessments, it underlines the importance of assessing all three muscle domains, especially in case of a clinical suspicion of secondary sarcopenia (7). Moreover, despite the limited number of patients included, our data indicate that patients with BC may be considered frail even in the absence of low muscle mass (4, 5). Further, the present data suggest that EWGSOP2 cut-off values might not be as suitable in detection of secondary sarcopenia. The EWGSOP2 definition, cut-off values and algorithm is developed to identify older people with sarcopenia where low muscle strength often precedes a decline in muscle mass. However, in patients with cancer (irrespective of age), the pathomechanics of muscle loss is mainly driven by the underlying disease, increased inflammation and/or chemotherapy treatment leading to secondary sarcopenia. Consequently, loss of muscle mass may very well precede a reduction in muscle strength and physical function. Moreover, cut-off values for primary sarcopenia may not apply very well to younger individuals. Compared to the prevalence of low muscle mass (55%–69%) in previous studies in patients with BC receiving NAC, this prevalence was considerably lower in our patient cohort (14). Notably however, as previously underlined by Simonsen and colleagues it is difficult to compare muscle mass assessed by DXA and CT, which hampers inter-study comparison and highlights the need for consensus within the field of sarcopenia (13).

The Covid-19 pandemic caused an extraordinary situation making conduction of the present study as well as many other Danish clinical studies impossible, as test facilities were decommissioned for months during spring 2020. The combination of a frail patient group, logistic challenges and a study requiring extra visits at the hospital with physically demanding tests in addition to the Covid-19 pandemic prevented a successful patient inclusion. In addition to difficulties recruiting patients, it proved challenging to conduct follow-up examinations, mainly due to logistic disadvantages and Covid-19. Difficulties in recruiting patients and performing trial examinations during the Covid-19 pandemic do not confine to medical oncology but are described across more medical and surgical specialties (15–20).

The failure to recruit and retain patients is the main limitation of this study. The original plan of analyses had to be abandoned and and a more descriptive approach was chosen in order not to overanalyze the sparse data material. Further, lack of data on patients eligible for study inclusion and, hence, on the recruitment rate is another study limitation. Despite these limitations, this study provides important information about the patient population and relevance of measurement methods which may be useful for future clinical studies. The study may therefore be considered a pilot study with correspondingly weighted scientific value.

## Conclusion

This prematurely terminated study illustrates the challenges of conducting clinical studies in frail patient groups. Our results indicate that a large part of patients with BC are affected in one or more muscle domains, with gait speed being the most frequently affected in the present study, however, no patients had sarcopenia according to EWGSOP2 guidelines.

## Data Availability

The raw data supporting the conclusions of this article will be made available by the authors, without undue reservation.
