# Supplementary material for: Muscle mass and physical function in patients with bladder cancer—Data from a prematurely terminated prospective cohort study
Source: Front Rehabil Sci. 2022 Oct 6;3:942475. doi: 10.3389/fresc.2022.942475 (PMC9582947; doi:10.3389/fresc.2022.942475)
Supplement: Supplementary file 1 [file DataSheet1.pdf]

Supplementary Table 1. Clinical characteristics of patients (n = 14) in the GESICA study, Copenhagen, 2018–2021.

	All (n = 14)<br>n (%)	Men (n = 8)<br>n	Women (n = 6)<br>n
**Age, years (range)**	74 (56–84)	75 (56–84)	72 (62–81)
**Primary tumor location**			
Renal pelvis/ureter	4 (29)	1	3
Bladder	10 (71)	7	3
Urethra	0 (0)	0	0
**Histology**			
Urothelial carcinoma	14 (100)	8	6
**Disseminated disease at diagnosis**			
Yes	4 (29)	3	1
No	10 (71)	5	5
**Prior treatments**			
NAC	0 (0)	0	0
Radical cystectomy	3 (21)	2	1
Nephroureterectomy	4 (29)	1	3
1L chemotherapy	1 (7)	1	0
**Location of metastases**			
Lymph nodes	8 (57)	6	2
Liver	1 (7)	0	1
Lungs	3 (21)	1	2
Bone	1 (7)	1	0
Pelvis soft tissue	3 (21)	0	3
Carcinosis	1 (7)	1	0
**ECOG PS**			
0	6 (43)	3	3
1	8 (57)	5	3
**Treatment initiated**			
NAC	4 (29)	2	2
1L cis/gem	2 (14)	2	0
1L carbo/gem	6 (43)	3	3
1L pembrolizumab	1 (7)	0	1
2L vinflunine	1 (7)	1	0

Abbreviations: ECOG PS = eastern cooperative oncology group performance status; NAC = neoadjuvant chemotherapy; 1L = first-line treatment; cis/gem = cisplatin/gemcitabine; carbo/gem = carboplatin/gemcitabine; 2L = second-line treatment.

Supplementary Table S2. Treatment details of patients (n = 14) in the GESICA study, Copenhagen, 2018-2021.

ID	Sex	Age	ECOG PS	Treatment completion≠ (%)	Reason for treatment termination	Dose reduction	Treatment delay
1	M	≥80	1	100	Treatment completed	Yes	No
2	F	70-79	1	100	Treatment completed	Yes	Yes
3	M	≥80	1	100	Treatment completed	Yes	Yes
4	F	60-69	1	50	Disease progression	Yes	No
5	M	70-79	0	100	Treatment completed	Yes	Yes
6	M	70-79	1	100	Treatment completed	Yes	Yes
7	F	60-69	0	100	Treatment completed	Yes	No
8	M	70-79	1	–*	Disease progression	Yes	Yes
9	M	60-69	0	100	Treatment completed	No	Yes
10	M	60-69	1	100	Treatment completed	Yes	Yes
11	F	60-69	0	25	Mental issues	No	No
12	M	50-59	0	75	Side effects	No	No
13	F	≥80	1	–∞	Disease progression	No	No
14	F	70-79	0	100	Treatment completed	Yes	Yes

Abbreviations: ECOG PS = eastern cooperative oncology group performance status; M = male; F = female.
≠ Regarded as completion of planned number of treatment cycles regardless of dose reductions and treatment delays.
*Patient received vinflunine with no defined maximum of treatment cycles (a total of six cycles received)
∞ Patient received pembrolizumab with no clear maximum of treatment cycles (a total of two cycles received)
